# Determinants of antibiotic prescriptions in a large cohort of children discharged from a pediatric emergency department

**DOI:** 10.1007/s00431-022-04386-y

**Published:** 2022-02-04

**Authors:** Marcello Covino, Danilo Buonsenso, Antonio Gatto, Rosa Morello, Antonietta Curatole, Benedetta Simeoni, Francesco Franceschi, Antonio Chiaretti

**Affiliations:** 1 Emergency Medicine, Fondazione Policlinico Universitario A. Gemelli, IRCCS, Rome, Italy; 2 Università Cattolica del Sacro Cuore, Rome, Italy; 3 Dipartimento Di Scienze Di Laboratorio E Infettivologiche, Fondazione Policlinico Universitario A. 8 Gemelli, IRCCS, Rome, Italy; 4 Department of Woman and Child Health and Public Health, Fondazione Policlinico Universitario 10 A. Gemelli, IRCCS, Rome, Italy; 5 Global Health Research Institute, Istituto Di Igiene, Università Cattolica del Sacro Cuore, Rome, Italy

**Keywords:** Antibiotics, Children, Emergency department

## Abstract

While there is evidence of high use of wide-spectrum antibiotics in children evaluated in the pediatric emergency departments, determinants of this behavior are still unclear. This study was aimed at defining the demographic, social, clinical, and laboratory factors that affect antibiotic prescriptions in children discharged from the emergency department. We performed a retrospective observational study of children aged younger than 18 years discharged from a pediatric university hospital between Jan. 1, 2015 and Dec. 31, 2020. We determined the proportion and type of antibiotic prescription according to demographic, social, clinical, laboratory, and imaging data, as well as doctor’s expertise. Fifty-one thousand six hundred thirty-three children were included, and 13,167 (25.5%) received an antibiotic prescription. Amoxicilline/clavulanate (Am/Cl) was the most prescribed antibiotic (8453, 64.2% of all prescriptions). Factors independently associated with an antibiotic prescription were older age (OR = 1.62 [1.53–1.73] for age 2–5 years, OR = 1.77 [1.64–1.91] for age 6–10 years, OR = 1.36 [1.25–1.49] for age 11–18 years, *p* < 0.001 for all groups); being evaluated by a physician with > 3 years of pediatric expertise (OR = 1.22 [1.13–1.31], *p* < 0.001); fever peak higher than 40 °C (OR = 1.37 [1.21–1.54], *p* < 0.001); abnormal findings on auscultation (OR = 1.95 [1.75–2.17], *p* < 0.001), CRP values (OR = 1.63 [1.26–2.10] for CRP < 50 mg/L, and OR = 3.78 (2.75–5.21) for CRP ≥ 50 mg/L with respect to CRP not requested; *p* < 0.01); CXR results whatever positive (OR = 4.47 [3.62–5.52], *p* < 0.001) or negative (1.82 [1.62–2.04], *p* < 0.001); being diagnosed with upper respiratory tract infections (OR = 4.27 [4.04–4.51], *p* < 0.001), lower respiratory tract infections (OR = 5.35 [4.88–5.85]; *p* < 0.001), and UTI (OR = 9.33 [8.14–10.71], *p* < 0.001).

*Conclusions*: Overprescription of antibiotics, including Am/Cl, is relevant in pediatric emergency departments. Factors associated with overprescription are not limited to the clinical characteristics of the treated patients. These findings highlight the need for a new and comprehensive approach to ensure successful antibiotic stewardship initiatives in the emergency departments.

**Table:** 

**What is Known:** *• Antibiotic resistance is a growing problem in medical practice, including in pediatrics.* *• Antibiotics are overprescribed in children assessed in the emergency department, but comprehensive and large studies are lacking.*	
**What is New:** *• Factors associated with overprescription are not limited to the clinical characteristics of the patients.* *• Non-clinical factors such as environmental variables, doctor’s expertise, and attitudes to laboratory and radiological examinations affect prescription.*

**Supplementary information:**

The online version contains supplementary material available at 10.1007/s00431-022-04386-y.

## Background

Antibiotic resistance is one of the biggest threats to global health, and, unfortunately, it is rising globally and is now at dangerous high levels. New resistance mechanisms are emerging and spreading, and a growing list of diseases, including the major global killers—such as pneumonia, tuberculosis, and foodborne diseases—are becoming more and more difficult to treat [1]. Recent estimates from the European Economic Area found that, in 2015, infections with antibiotic-resistant bacteria accounted for an estimated 33,110 (28,480–38,430) attributable deaths and 874,541 (768,837–989,068) disability-adjusted life-years (DALYs) [2], and had increased since 2007. Unfortunately, this problem is increasingly recognized in the pediatric population as well, with the highest burden in infants aged < 1 year [2].

Tackling antibiotic resistance is challenging since it requires integrated and multifactorial interventions targeted at all levels of society, from the daily habits of individuals to the healthcare industry, from policymakers to health professionals. In this complex scenario, appropriate antibiotic prescription practices play an obvious role and, at least theoretically, represent the easiest area of intervention. Understanding reasons behind physicians’ choices to prescribe antibiotics and how improving these practices represent a priority for modern medicine.

In this context, children are a well-known and recognized target of antibiotic prescriptions [3], making them the category that most uses antibiotics [4]. In particular, primary care and emergency departments (EDs) are settings where overprescription seems particularly high. The latter, in particular, is a very specific context. Legal risks, lack of access to comprehensive diagnostics and clinical background, high flows of patients, lack of dedicated follow-up paths (safety nets), and difficulties in objective and safe distinctions between viral and bacterial infections make this setting particularly prone to overprescription [5]. However, addressing all these factors may be challenging. Although several studies tried addressing the antibiotic prescription in pediatric emergency departments, none made a comprehensive approach.

Most of these studies were single center and included small numbers [6], and the few multinational ones considered a random sampling of patients on specific days of the week [5, 7]. Other studies only included feverish children or pneumonia, excluding several potential presentations which, indeed, could be the still receive antibiotic prescriptions [5, 8]. Moreover, physicians’ expertise, or even seasonality, or daytime of ED access are factors that may affect prescription practices and are usually not considered in the available research. Finally, patient’s characteristics such as specific comorbidities, or the decision to perform assessments rather than the simple result, are rarely included in these studies.

For these reasons, to fill the current knowledge gap, we designed this study aimed to comprehensively assess the factors associated with the antibiotic prescriptions in children discharged from the ED of a University Hospital, including several variables not yet assessed in current literature.

## Methods

### Study design and participants

This is a retrospective cross-sectional observational study conducted in the pediatric ED of the Fondazione Policlinico Universitario A. Gemelli IRCCS of Rome, Italy, which is a third-level ED and a regional trauma center. Our ED held a dedicated pediatric section, with a senior pediatrician present 24/day, in addition to one or more residents (pediatrician in training). The final disposition, the medical responsibility, and signature on the discharge letters are always made by the senior pediatrician.

We included in the analysis all children aged ≤ 17 years who accessed the pediatric ED between Jan. 1, 2015 and Dec 31, 2020, and were discharged with suspect or confirmed infectious disease.

We excluded from the analysis the patients not discharged directly from the ED (including those admitted in the pediatric wards, in the Pediatric Intensive Care Unit, and those transferred to other hospitals). We also excluded patients assessed for any type of trauma, or post-surgery complications, since they may have received antibiotic prescriptions for prophylaxis. Moreover, since the study period has partial overlap with the COVID-19 pandemic, we excluded children with SAR-CoV-2 infection that, particularly during the first months of uncertainties, may have influenced the doctor’s choice of prescribing antibiotics. Since the beginning of the pandemic, children with signs or symptoms suggestive of an infectious disease were tested with PCR test on nasopharyngeal swab, and children with a positive result were excluded. Finally, we excluded patients with inconsistent or incomplete medical records.

This study was conducted according to the principles expressed in the declaration of Helsinki and its later amendments and was approved by the local Institutional Review Board (ID 3497, Prot 0,013,703/21).

### Procedures

Data were retrospectively collected from the computerized clinical records of our institution, and included in an electronic database.

Collected variables included the following:

- Characteristics of ED access, including season, day, and daytime of the ED access; self-reported or transferred by the emergency medical system; triage code assigned.
- The expertise of the treating pediatrician (cathegorized as > 3 years or lower).
- Patients’ demographic, including age and sex.
- Reported symptoms, including cough, vomit, dyspnea, diarrhea, fever peak > 40 °C, myalgia/arthralgia, palpitations, and headache
- Clinical findings at ED evaluation, including fever > 37.5 °C, crepitation on auscultation, poor or mediocre general condition, peripheral cyanosis (including capillary refill > 2 s).
- Clinical history, including the presence of major inherited disease (any), history of cardiac disease, history of prematurity, history of asthma, intellectual disability, history of epilepsy.
- Laboratory evaluation including WBC count (if obtained), PCR value (if obtained), urine analysis (if obtained). Laboratory tests were available 24 h/day in our institution in the study period. A positive WBC count was considered WBC > 10^9^ cell/L. A positive CRP was considered for CRP ≥ 50 mg/L. For CRP, we chose a 50 mg/dL cutoff since available literature describes it as the most realistic to discriminate bacterial and viral infections [9] and, therefore, may theoretically influence pediatrician decisions to prescribe antibiotics. According to the Italian Guidelines, we considered a positive urine analysis in case of both nitrite leucocyte esterase-positive results (infection very likely according to the guidelines) or leucocyte esterase-positive results (infection likely according to the guidelines) [10]. About diagnostics, we focused on these tests since they are the most frequently prescribed and available tests in the general pediatric ED setting. For the same reason, we did not include in the analysis the procalcitonin value, since this latter test was only recently implemented as a routine test in our institution and is not generally available in all EDs.
- Chest X-ray (CRX) results (if obtained). Radiology was available 24 h/day in our institution in the study period. A positive CRX was defined as the presence of any pulmonary infiltrate or lesion compatible with an infective diagnosis. The CRX evaluation was obtained by the radiologist’s reports.
- ED discharge diagnosis. The discharge diagnosis was obtained by the ICD9 code of discharge matched with the textual diagnosis in the clinical charts. In the case of multiple infective diagnoses, only the primary diagnosis was considered. The infective diagnoses were grouped in upper airway infections (including nose, ear, and throat infections), lower airway infections (including pneumonia and bronchitis), abdominal infections (including gastroenteritis, mesenteritis, and abdominal conditions like appendicitis), urinary tract infections, cutaneous and follicular infections, other specified infections (including generalized infections like scarlet fever, other common pediatric exanthema infections), and unspecified fever (including unconfirmed suspect viral infections).
- Prescription of any antibiotic at discharge. The antibiotic prescriptions were grouped for further analysis in amoxicillin, amoxicillin/clavulanic (Am/Cl), oral cephalosporin, injective cephalosporin, clarithromycin, azithromycin, fluoroquinolone, trimethoprim, others.

We used the Strengthening the Reporting of Observational Studies in Epidemiology (STROBE) guidelines to report this study.

### Outcome measures

The primary outcome measure was the presence of an antibiotic prescription at the discharge from the pediatric ED.

Secondary outcome measures were as follows:

- The prescription of Am/Cl at discharge (since current literature agrees with this being the most used antibiotic in the pediatric population, despite its wider spectrum and guidelines favor amoxicillin alone for most conditions) [3].
- The prescription of amoxicillin alone at discharge.

### Statistical analyses

Continuous variables were reported as median [interquartile range], and are compared at univariate analysis by Mann–Whitney *U* test or Kruskal–Wallis test in case of three or more groups. Categorical variables were reported as absolute number (percentage), and are compared by chi-squared test (with Fisher’s test if appropriate).

Variables having a significant association with an antibiotic prescription at discharge from the ED were entered into a logistic regression model in order to identify independent predictors of prescription. Prior to being entered into the logistic models, for a better model fitting and odds estimation, we transformed the continuous variables age, WBC, and PCR into categorical variables. For age, patients were divided into age < 1-year-old, age 2–5 years, age 6–10 years, and age 11–18 years; the reference category for odds calculation was the group age < 1 year. For WBC, patients were divided into “WBC not requested,” WBC < 10^3^/mm^3^, and WBC ≥ 10^3^/mm^3^; the reference category for odds calculation was considered WBC not requested. For PCR, patients were divided into “PCR not requested,” PCR < 50 mg/dL, and PCR ≥ 50 mg/dL; the reference category for odds calculation was considered PCR not requested.

To avoid model redundancy or overfitting, single items composing derived variables were excluded from multivariate analysis,

The univariate and multivariate analysis was performed considering the whole population at the same time, and the single age group separately. Similarly, multiple models were obtained for “general antibiotic prescription,” Am/Cl prescription, and single amoxicillin prescription.

Multivariate association of factors with the study endpoints was expressed as odds ratio (OR) [95% confidence interval]. We considered significant a two-sided *p* ≤ 0.05. Data were analyzed by SPSS v25® (IBM, Armonk, NY, USA).

## Results

### Study population

In the study period, 112,516 patients ≤ 17 years were evaluated in the pediatric ED of our institution. Considering the inclusion criteria, 51,633 children discharged with suspect or confirmed infective diagnosis were included in the analyses (Table 1).

**Table 1:** Demographic and clinical characteristics and antibiotic prescriptions of the study population

**Variable**	**All patients**	** < 1 year old**	**2–5 years old**	**6–10 years old**	** > 10 years old**
	***N*** **51,633**	***N*** **8,982**	***N*** **26,516**	***N*** **9,724**	***N*** **6,411**
Sex (male)	28,384 (55.0)	5043 (56.1)	14,844 (56.0)	5240 (53.9)	3257 (50.8)
Age (years)	3 [1–6]	8 [5–10]	2 [2–4]	7 [6–8]	13 [11–15]
Ed presentation					
Triage code					
- Emergency	43 (0.1)	2 (0.0)	31 (0.1)	5 (0.1)	5 (0.1)
- Urgent	2878 (5.7)	615 (7.0)	1263 (4.9)	449 (4.7)	2878 (5.7)
- Non-urgent	47,152 (93.7)	8120 (92.7)	24,584 (94.6)	8952 (94.5)	47,152 (93.7)
- Ambulatory	264 (0.5)	19 (0.2)	96 (0.4)	67 (0.7)	264 (0.5)
Access on weekend	19,784 (38.5)	3426 (38.1)	10,628 (40.1)	3730 (38.4)	2090 (32.6)
Season of ED access					
- Summer	8672 (16.8)	1413 (15.7)	3919 (14.8)	1686 (17.3)	1654 (25.8)
- Fall	12,282 (23.8)	2112 (23.5)	6819 (25.7)	2000 (20.6)	1351 (21.1)
- Winter	17,491 (33.9)	3346 (37.3)	8996 (33.9)	3316 (34.1)	1833 (28.6)
- Spring	13,188 (25.5)	2111 (23.5)	6782 (25.6)	2722 (28.0)	1573 (24.5)
Access on nightshift	24,977 (48.4)	4378 (48.7)	12,997 (49.0)	4555 (46.8)	3047 (47.5)
Access by EMS	1263 (2.4)	173 (1.9)	747 (2.8)	169 (1.7)	174 (2.7)
Pediatrician expertise > 3 years	46,055 (89.2)	8243 (91.8)	24,410 (92.1)	8822 (90.7)	4580 (71.4)
Reported symptoms					
Cough	15,482 (30.0)	3596 (40.0)	8418 (31.7)	2332 (24.0)	1136 (17.7)
Vomit	10,218 (19.8)	1674 (18.6)	5331 (20.1)	2087 (21.5)	1126 (17.6)
Dyspnea	2015 (3.9)	738 (8.2)	856 (3.2)	227 (2.3)	194 (3.0)
Diarrhea	6211 (12.0)	1261 (14.0)	3448 (13.0)	856 (8.8)	646 (10.1)
Fever peak > 40 °C	1596 (3.1)	278 (3.1)	919 (3.5)	221 (2.3)	178 (2.8)
Myalgia/arthralgia	165 (0.3)	2 (0.0)	40 (0.2)	53 (0.5)	70 (1.1)
Palpitations	104 (0.2)	12 (0.1)	27 (0.1)	25 (0.3)	40 (0.6)
Headache	1734 (3.4)	0	371 (1.4)	733 (7.5)	630 (9.8)
Clinical evaluation					
Fever > 37.5 °C on admission	40,974 (79.4)	7258 (80.8)	22,215 (83.8)	7094 (73.0)	4407 (68.7)
Crepitation on auscultation	1988 (3.9)	481 (5.4)	1057 (4.0)	337 (3.5)	113 (1.8)
Poor or mediocre general condition	652 (1.3)	124 (1.4)	352 (1.3)	102 (1.0)	74 (1.2)
Peripheral cyanosis	387 (0.7)	85 (0.9)	185 (0.7)	63 (0.6)	54 (0.8)
Laboratory and radiology					
CRP mg/l	12.1 [2.1–34.1]	8.2 [2.0–22.4]	17.1 [5.3–42.8]	11.9 [2.1–33.3]	9.2 [0.6–31.3]
CRP categoric					
- Not requested	48,667 (94.3)	8578 (95.5)	25,524 (96.3)	9109 (93.7)	5456 (85.1)
- < 50 mg/L	2466 (4.8)	357 (4.0)	785 (3.0)	520 (5.3)	804 (12.5)
- ≥ 50 mg/L	500 (1.0)	47 (0.5)	3.8(0.8)	95 (1.0)	151 (2.4)
WBC cell/mm^3^	9.6 [7.1–13.2]	10.8 [7.7–14.2]	10.6 [7.7–14.5]	9.1 [6.8–12.7]	8.8 [6.9–11.6]
WBC categoric					
- Not requested	48,590 (94.1)	8584 (95.6)	25,502 (96.2)	9093 (93.5)	5411 (84.4)
- < 10 k cell/mm^3^	1645 (3.2)	183 (2.0)	463 (1.7)	371 (3.8)	628 (9.8)
- ≥ 10 k cell/mm^3^	1398 (2.7)	215 (2.4)	551 (2.1)	260 (2.7)	372 (5.8)
Urine analysis					
- Not requested	51,147 (99.1)	8847 (98.5)	26,353 (99.4)	9659 (99.3)	6288 (98.1)
- Negative	332 (0.6)	105 (1.2)	114 (0.4)	39 (0.4)	74 (1.2)
- Positive	154 (0.3)	30 (0.3)	26 (0.3)	49 (0.8)	49 (0.8)
Chest X-ray					
- Not requested	49,371 (95.6)	8724 (97.1)	25,392 (95.8)	9285 (95.5)	5970 (93.1)
- Negative	1642 (3.2)	213 (2.4)	805 (3.0)	271 (2.8)	353 (5.5)
- Positive	620 (1.2)	45 (0.5)	319 (1.2)	168 (1.7)	88 (1.4)
Clinical history					
Inherited disease	287 0.6)	40 (0.4)	127 (0.5)	52 (0.5)	68 (1.1)
History of cardiac disease	33 (0.1)	6 (0.1)	8 (0.0)	6 (0.1)	13 (0.2)
Prematurity	281 (0.5)	111 (1.2)	136 (0.5)	24 (0.2)	10 (0.2)
History of asthma	1475 (2.9)	181 (2.0)	779 (2.9)	317 (3.3)	198 (3.1)
Intellectual disability	42 (0.1)	0	15 (0.1)	20 (0.2)	7 (0.1)
History of epilepsy	189 (0.4)	17 (0.2)	98 (0.4)	44 (0.5)	30 (0.5)
Diagnostic group					
Upper airway infections	21,409 (41.5)	3408 (37.9)	12,288 (46.3)	3809 (39.2)	1904 (29.7)
Lower airway infections	4699 (9.1)	1455 (16.2)	2119 (8.0)	779 (8.0)	346 (5.4)
Abdominal	4314 (8.4)	455 (5.1)	1726 (6.5)	1153 (11.9)	980 (15.3)
Urinary tract infections	1125 (2.2)	213 (2.4)	396 (1.5)	241 (2.5)	275 (4.3)
Cutaneous	1494 (2.9)	189 (2.1)	733 (2.8)	331 (3.4)	241 (3.8)
Other specified	1053 (2.0)	525 (2.0)	249 (2.6)	232 (3.6)	1053 (2.0)
Fever (unspecified)	17,539 (34.0)	3215 (35.8)	8729 (32.9)	3162 (32.5)	2433 (38.0)
Prescription at discharge					
Antibiotic (any)	13,167 (25.5)	1802 (20.1)	7341 (27.7)	2672 (27.5)	1352 (21.1)
Amoxicillin	1909 (3.7)	495 (5.5)	1144 (4.3)	223 (2.3)	47 (0.7)
Amoxicillin/clavulanic	8453 (16.4)	997 (11.1)	4701 (17.7)	1807 (18.6)	948 (14.8)
Azitromicin	112 (0.2)	7 (0.1)	54 (0.2)	31 (0.3)	36 (0.6)
Cefaclor	1129 (2.2)	131 (1.5)	724 (2.7)	225 (2.3)	49 (0.8)
Ceftriaxone	180 (0.3)	8 (0.1)	59 (0.2)	52 (0.5)	61 (1.0)
Claritromicine	1199 (2.3)	162 (1.8)	614 (2.3)	286 (2.9)	137 (2.1)
Other	169 (0.3)	2 (0.0)	45 (0.2)	48 (0.5)	74 (1.2)

Their median age was 3 years (IQR 1–6), and 28,384 (55%) were males. Two thousand eight hundred eighty-one (5.8%) children were triaged as emergent or urgent cases (Table 1).

Overall, one in four discharged children (25.5%) received an antibiotic prescription, with Am/Cl being the most prescribed antibiotic (8.453 patients, 64.2% of all prescriptions). Further details on the study population are reported in Table 1.

### Determinants of antibiotic prescriptions

Table 2 shows factors associated or not with a higher rate of antibiotic prescription at discharge. Overall, older age (*p* < 0.001), winter season (*p* < 0.001), being assessed by a more experienced pediatrician (*p* < 0.001), having inherited (*p* < 0.001), or cardiac diseases (*p* = 0.026) were the demographic/anamnestic factors associated with a higher antibiotic prescription. Other clinical and laboratory factors associated or not to antibiotic prescriptions on univariate analyses are detailed on Table 1.

**Table 2:** Univariate and multivariate analyses of factors associated with antibiotic prescriptions

**Variable**	**No antibiotic prescription (*n*** **38,466)**	**Antibiotic prescription **(***n*** **13,167)**	***n*** **value**	**Odds ratio (95% confidence interval)**	**Multivariate** ***p* value**
Sex (male)	21,057 (54.7)	7327 (55.6)	0.072		
Age (years)	3 [1–6]	3 [2–6]	** < 0.001**	/	
Age group				/	
- Age < 1 year	7180 (18.7)	1802 (13.7)		Reference	
- Age 2–5 years	19,175 (49.8)	7341 (55.8)	** < 0.001**	1.62 (1.53–1.73)	< 0.001
- Age 6–10 years	7052 (18.3)	2672 (20.3)		1.77 (1.64–1.91)	< 0.001
- Age 11–18 years	5059 (13.2)	1352 (10.3)]		1.36 (1.25–1.49)	< 0.001
Ed presentation					
Triage code					
- Emergency	31 (0.1)	12 (0.1)			
- Urgent	2125 (5.7)	753 (5.8)	0.144		
- Non-urgent	35,036 (93.7)	12,116 (93.7)			
- Ambulatory	212 (0.6)	52 (0.4)			
Access on weekend	14,803 (38.5)	5071 (38.5)	0.952		
Season of ED access					
- Summer	6502 (16.9)	2170 (16.5)		Reference	
- Fall	9185 (23.9)	3097 (23.5)	**0.001**	0.93 (0.87–1.00)	
- Winter	12,841 (33.4)	4650 (35.3)		1.03 (0.96–1.09)	0.438
- Spring	9938 (25.8)	3250 (24.7)		0.98 (0.91–1.05)	0.564
Access on nightshift	19,106 (49.7)	5871 (44.6)	** < 0.001**	0.83 (0.79–0.87)	< 0.001
Access by EMS	938 (2.4)	325 (2.5)	0.849		
Pediatrician expertise > 3 years	34,086 (88.6)	11,969 (90.9)	** < 0.001**	1.22 (1.13–1.31)	< 0.001
Reported symptoms					
Cough	10,908 (28.4)	4574 (34.7)	** < 0.001**	0.98 (0.94–1.03)	0.527
Vomit	8359 (21.7)	1859 (14.1)	** < 0.001**	0.85 (0.80–0.91)	< 0.001
Dyspnea	1559 (4.1)	456 (3.5)	0.003	0.24 (0.48–0.61)	< 0.001
Diarrhea	5214 (13.6)	997 (7.6)	** < 0.001**	1.04 (0.96–1.13)	0.31
Fever peak > 40 °C	1089 (2.8)	507 (3.9)	** < 0.001**	1.37 (1.21–1.54)	< 0.001
Myalgia/arthralgia	143 (0.4)	22 (0.2)	** < 0.001**	0.60 (0.37–0.97)	0.039
Palpitations	77 (0.2)	27 (0.2)	0.914		
Headache	1383 (3.6)	351 (2.7)	** < 0.001**	0.87 (0.76–0.99)	0.036
Clinical evaluation					
Fever > 37.5 °C on admission	30,369 (79.0)	10,605 (80.5)	** < 0.001**	1.26 (1.11–1.42)	< 0.001
Crepitation on auscultation	946 (2.5)	1042 (7.9)	** < 0.001**	1.95 (1.75–2.17)	< 0.001
Poor or mediocre general condition	439 (1.1)	213 (1.6)	** < 0.001**	1.08 (1.03–1.13)	< 0.001
Peripheral cyanosis	291 (0.8)	96 (0.7)	0.753		
Laboratory and radiology					
CRP mg/l	8.7 [1.0–25.3]	23.5 [9.7–56.4]	** < 0.001**	/	
CRP categoric					
- Not requested	36,384 (94.6)	12,283 (93.3)		Reference	
- < 50 mg/L	1829 (4.8)	637 (4.8)	** < 0.001**	1.63 (1.26–2.10)	< 0.001
- ≥ 50 mg/L	253 (0.7)	247 (1.9)		3.78 (2.75–5.21)	0.013
WBC cell/mm^3^	9.1 [6.9–12.3]	11.4 [8.1–14.9]	** < 0.001**	/	
WBC categoric					
- Not requested	36,293 (94.4)	12,297 (93.4)		Reference	
- < 10 k cell/mm^3^	1291 (3.4)	354 (2.7)	** < 0.001**	0.71 (0.54–0.93)	0.035
- ≥ 10 k cell/mm^3^	882 (2.3)	516 (3.9)		1.15 (0.87–1.50)	0.086
Urine analysis					
- Not requested	38,150 (99.2)	12,997 (98.7)		Reference	
- Negative	260 (0.7)	72 (0.5)	** < 0.001**	1.30 (0.96–1.76)	< 0.001
- Positive	56 (0.1)	98 (0.7)		1.42 (0.99–2.04)	< 0.001
Chest X-ray					
- Not requested	37,390 (97.2)	11,981 (91.0)	** < 0.001**	Reference	
- Negative	949 (2.5)	693 (5.3)		1.82 (1.62–2.04)	< 0.001
- Positive	127 (0.3)	493 (3.7)		4.47 (3.62–5.52)	< 0.001
Clinical history					
Inherited disease	187 (0.5)	100 (0.8)	** < 0.001**	1.05 (0.96–1.28)	0.439
History of cardiac disease	19 (0.0)	14 (0.1)	**0.026**	0.97 (0.44–1.15)	0.521
Prematurity	201 (0.5)	80 (0.6)	0.252		
History of asthma	1087 (2.8)	388 (2.9)	0.472		
Intellectual disability	30 (0.1)	12 (0.1)	0.648		
History of epilepsy	135 (0.4)	54 (0.4)	0.332		
Diagnostic group					
Upper airway infections	13,904 (36.1)	7505 (57.0)		4.27 (4.04–4.51)	< 0.001
Lower airway infections	2480 (6.4)	2219 (16.9)		5.35 (4.88–5.85)	< 0.001
Abdominal	4232 (11.0)	82 (0.6)		0.14 (0.11–0.18)	< 0.001
Urinary tract infections	508 (1.3)	617 (4.7)	** < 0.001**	9.33 (8.14–10.71)	< 0.001
Cutaneous	911 (2.4)	583 (4.4)		4.93 (4.39–5.54)	< 0.001
Other specified	897 (2.3)	156 (1.2)		1.28 (1.06–1.53)	0.007
Fever (unspecified)	15,534 (40.4)	2005 (15.2)		Reference	

When entered into a multivariate logistic regression model, several factors emerged as independent predictors of antibiotic prescription (Table 2).

Clinical findings and symptoms independently associated with an antibiotic prescription were a reported fever peak > 40 °C (OR 1.37), the presence of fever > 37.5 °C at the physical examination (OR 1.26, *p* < 0.001), a poor or mediocre general condition (OR 1.08, *p* < 0.001), and positive thorax auscultation (OR 1.95, *p* < 0.001).

A PCR ≥ 50 mg/dL, a positive urine analysis, and a positive CXR were independently associated with an antibiotic prescription at ED discharge. Conversely, the sole prescription of these examinations in the ED was associated with a higher odds of antibiotic prescription (Table 2), compared to patients discharged with no examination. Pediatricians with > 3-year experience showed a significantly higher attitude to antibiotic prescription (OR 1.22, *p* < 0.001) compared to less experienced colleagues. Other factors are reported in Table 2.

Multivariate analyses of factors influencing the antibiotic prescription for each age group are presented in Supplementary Table 1.

### Types of antibiotic prescriptions

**Fig. 1:**
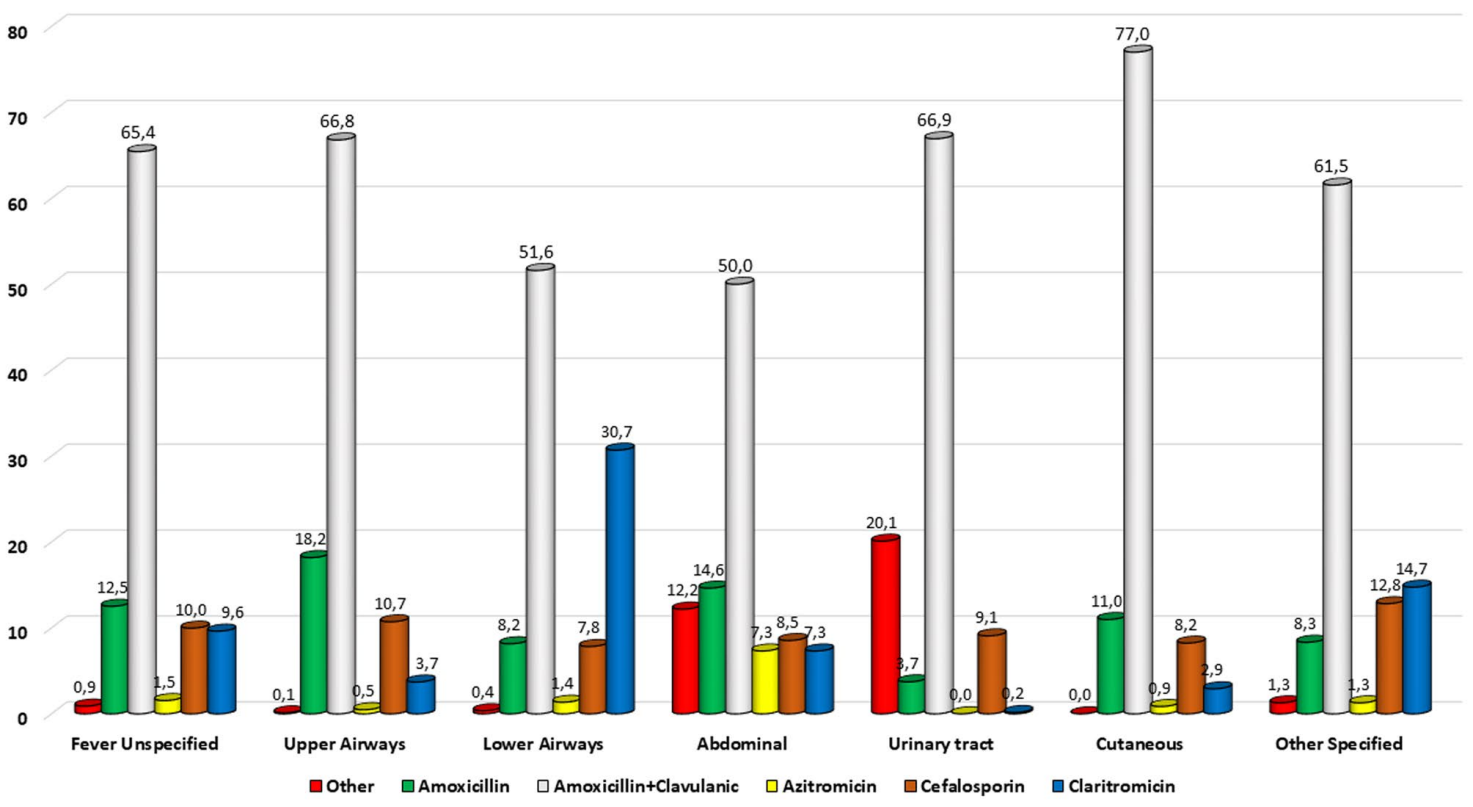
Pattern of antibiotic prescription according to the main discharge diagnosis. The percentage was calculated on the 13,197 patients discharged with an antibiotic prescription

The antibiotics prescribed according to the discharge diagnoses are represented in Fig. 1. In each of the main diagnostic categories, Am/Cl was always the most prescribed antibiotic. Details about each antibiotic prescribed by diagnostic group and age group are reported in the Supplementary Figures SF-1 to SF-4.

In each age group, Am/Cl remained largely the most prescribed antibiotic, although use of macrolides and other drugs like fluoroquinolones was higher in older age groups.

Table 3 shows the univariate and multivariate analyses of factors influencing the prescription of Am/Cl, instead of other active principles.

**Table 3:** Univariate and multivariate analyses of factors associated with prescription of amoxicilline/clavulanate. Analyses conducted on the subgroup of 13,167 children that received an antibiotic prescription

**Variable**	**Other antibiotic (*n*** **4698)**	**Amoxicillin + clavulanic (*n*** **8469)**	**Univariate**	**Odds ratio for prescription**	**Multivariate** ***p*** **value**
			***p*** **value**		
Sex (male)	2582 (55.0)	4745 (56.0)	0.237		
Age group					
- < 1 year	805 (17.1)	997 (11.8)		Reference	
- 2–5 years	2633 (56.0)	4798 (55.6)	** < 0.001**	1.38 [1.25–1.54]	< 0.001
- 6–10 years	859 (18.3)	1813 (21.4)		1.59 [1.40–1.81]	< 0.001
- 11–18 years	401 (8.5)	951 (11.2)		1.72 [1.47–2.01]	< 0.001
Ed presentation					
Triage code					
- Emergency	7 (0.2)	5 (0.1)			
- Urgent	292 (6.3)	461 (5.5)	0.082		
- Non-urgent	4291 (93.1)	7825 (94.0)			
- Ambulatory	21 (0.5)	31 (0.4)			
Access on weekend	1720 (36.6)	3351 (39.6)	**0.001**	1.09 [1.01–1.17]	0.023
Season of ED access					
- Summer	713 (15.2)	1457 (17.2)		Reference	
- Fall	1073 (22.8)	2024 (23.9)	**0.002**	1.08 [0.95–1.26]	0.224
- Winter	1736 (37.0)	2914 (34.4)		0.99 [0.88–1.11]	0.874
- Spring	1176 (25.0)	2074 (24.5)		0.93 [0.83–1.05]	0.286
Access on nightshift	2041 (43.4)	3830 (45.2)	**0.051**		
Access by EMS	92 (2.0)	233 (2.8)	**0.005**	1.41 [1.09–1.80]	0.008
Pediatrician expertise > 3 years	4173(88.8)	7796 (92.1)	** < 0.001**	1.46 [1.29–1.65]	< 0.001
Reported symptoms					
Cough	2018 (43.0)	2556 (30.2)	** < 0.001**	0.69 [0.64–0.76]	< 0.001
Vomit	693 (14.8)	1166 (13.8)	0.121		
Dyspnea	223 (4.7)	233 (2.8)	** < 0.001**	0.85 [0.70–1.04]	0.127
Diarrhea	403 (8.6)	594 (7.0)	**0.001**	0.88 [0.77–1.01]	0.077
Fever peak > 40 °C	160 (3.4)	347 (4.1)	**0.048**	1.23 [1.01–1.50]	0.035
Myalgia/arthralgia	4 (0.1)	18 (0.2)	0.086		
Palpitations	10 (0.2)	17 (0.2)	0.883		
Headache	81 (1.7)	270 (3.2)	** < 0.001**	1.54 [1.19–1.98]	0.001
Clinical evaluation					
Fever > 37.5 °C on admission	3859 (82.1)	6746 (79.7)	**0.001**	0.99 [0.89–1.09]	0.842
Crepitation on auscultation	477 (10.2)	565 (6.7)	** < 0.001**	1.01 [0.87–1.17]	0.91
Poor or mediocre general condition	87 (1.9)	126 (1.5)	0.113		
Peripheral cyanosis	20 (0.4)	76 (0.9)	**0.002**	2.05 [1.20–3.39]	0.005
Laboratory and radiology					
PCR categoric					
- Not requested	4375 (93.1)	7908 (93.4)			
- < 50 mg/L	237 (5.0)	400 (4.7)	0.689		
- ≥ 50 mg/L	86 (1.8)	161 (1.9)			
WBC categoric					
- Not requested	4390 (93.4)	7907 (93.4)			
- < 10 k cell/mm^3^	132 (2.8)	222 (2.6)	0.62		
- ≥ 10 k cell/mm^3^	176 (3.7)	340 (4.0)			
Urine analysis					
- Not requested	4651 (99.0)	8346 (98.5)			
- Negative	20 (0.4)	52 (0.6)	0.089		
- Positive	27 (0.6)	71 (0.8)			
Chest X-ray					
- Not requested	4159 (88.5)	7822 (92.4)		Reference	
- Negative	371 (7.9)	322 (3.8)	** < 0.001**	0.68 [0.58–0.81]	< 0.001
- Positive	168 (3.6)	325 (3.8)		1.92 [1.55–1.38]	< 0.001
Clinical history					
Inherited disease	40 (0.9)	60 (0.7)	0.365		
History of cardiac disease	1 (0.0)	13 (0.2)	**0.026**	8.62 [1.16–66.6]	0.035
Prematurity	35 (0.7)	45 (0.5)	0.131		
History of asthma	185 (3.9)	203 (2.4)	** < 0.001**	0.79 [0.64–0.98]	0.039
Intellectual disability	4 (0.1)	8 (0.1)	0.865		
History of epilepsy	21 (0.4)	33 (0.4)	0.622		
Diagnostic group					
Upper airway infections	2492 (53.0)	5013 (59.2)		1.02 [0.91–1.13]	0.733
Lower airway infections	1074 (22.9)	1145 (13.5)		0.66 [0.57–0.76]	< 0.001
Abdominal	41 (0.9)	41 (0.5)		0.47 [0.30–0.75]	0.001
Urinary tract infections	204 (4.3)	413 (4.9)	** < 0.001**	0.94 [0.77–1.15]	0.575
Cutaneous	134 (2.9)	449 (5.3)		1.51 [1.22–1.89]	< 0.001
Other specified	60 (1.3)	96 (1.1)		0.77 [0.55–1.09]	0.981
Fever (unspecified)	693 (14.8)	1312 (15.5)		Reference	

Compared to patients < 1 year, the older age groups had significantly higher odds of Am/Cl prescription, being the overall OR almost doubled in the 11–17-year group.

The access on weekends, on nightshifts, the transportation by EMS, and higher pediatrician expertise were all associated with higher odds of Am/Cl prescription.

The clinical findings independently associated with Am/Cl prescription were the presence of cough, a fever peak > 40 °C, a positive CRX, the presence of headache, and a history of cardiac disease. As expected, Am/Cl had a higher odds of prescription (compared to other classes of antibiotics) in cutaneous infections.

The multivariate analyses of factor influencing the prescription of amoxicillin, the antibiotic expected to be theoretically more prescribed, is presented in the Supplementary Table S2.

## Discussion

In this study, we provide a comprehensive overview of prescriptions and their determinants in a large sample of children assessed in the pediatric ED. To our knowledge, this is the largest study that also includes a comprehensive list of non-clinical variables that may affect antibiotic prescription, adding to patients’ signs and symptoms, and to a wide range of diagnostic categories, the daytime of presentation, the seasonality, the physicians’ expertise, and the physicians’ attitude for diagnostic request.

Overall, we found that a large proportion of children, as high as one in four children, were discharged from the ED with antibiotics. Among the antibiotic classes, Am/Cl was the most prescribed, representing more than half of all prescriptions. These data are in line with previous studies from Europe and the USA [5, 7, 11–13], and despite that our cohort is more recent, the evidence of overprescription of antibiotics in pediatric ED is still a major and unsolved problem. A recent large survey of Italian parents confirmed that Am/Cl was the most used antibiotic [3]. Despite that the majority of guidelines suggest amoxicillin as a first-line treatment for pharyngitis [14], pneumonia [15], and otitis media [16], Am/Cl was the most prescribed in all age groups and discharge diagnostic categories. This happens despite that it is well known that the large majority of pediatric infections are of viral origin and that even pediatric pneumonia rarely benefits from antibiotics compared with placebo [17, 18].

Contrarily to adults, the presence of pre-existing comorbidities did not seem to significantly affect antibiotic prescriptions in this pediatric cohort. However, those with inherited and cardiac diseases could have a higher odds of receiving antibiotics, and other fragile children such as ex-premature did not have this risk. However, it is indeed possible that these children were more frequently admitted to the ward rather than discharged, thus limiting the analysis for this group of children. This is confirmed by a multinational European study showing that children with inherited comorbidities are more frequently admitted by pediatric EDs [5].

Distinguishing bacterial from viral infections is challenging, particularly in the EDs, and the newer and promising transcriptomic tests are not available in routine settings. Moreover, the traditional dichotomous distinction of viral and bacterial has been recently challenged by the PERFORM consortium, which proposed a novel framework for phenotyping children with suspected or confirmed bacterial/viral infections [19]. However, while awaiting for new advances to be applied in real practice, some routine blood biomarkers such as CRP are traditionally used as first-line tests to support decision rules to discriminate between the two main groups. Although there is much variability and overlap, main studies suggest that CRP concentrations of < 5 mg/L (50 mg/L according to our assays) can effectively rule out SBI (likelihood ratio 0.087, 95% CI 0.02–0.38) [9]. Therefore, we chose this cutoff for our analyses. However, although those that received antibiotics had higher median levels of CRP and the likelihood increased with increasing values, a large proportion of children discharged with antibiotics had vales < 50 mg/L. In particular, an interesting finding was that, when the doctor decided not to perform CRP, children had already a lower probability of receiving antibiotics. The same happened with CBC count, urinalyses, and CXR. This approach is particularly evident with CXR, which were associated with significantly higher prescription independently from the evidence of pathological findings. Findings were very similar concerning urinalyses. These findings suggest that the doctor’s feeling of a more severe disease, which required further investigations, is itself a determinant of the final choice of antibiotic prescription, probably more important than the result itself. Similar behavior was recently found in smaller US and European cohorts of children assessed for suspected pneumonia [20–22], for which the doctor’s intention to treat was a more important factor for antibiotic prescription compared with the CXR results.

Clinical signs and symptoms can, indeed, help clinicians in treatment decisions. We found that some symptoms that may be more suggestive of viral infections, such as myalgias/arthralgias, gastrointestinal symptoms, and dyspnea (which is more frequent in children with wheezing or asthma, more frequently linked with viral infections), were associated with a lower likelihood of receiving antibiotics. However, the relevant number of children with signs or symptoms and laboratory investigations suggestive of viral infection that received antibiotics suggests a wide variability among physicians and the possibility that other factors may affect the prescription decision.

For these reasons, we also investigated non-clinical factors, such as the timing of the ED access and the day of the week (weekend vs working days). In our country, general practitioners are not available during the night and the weekends, nor routine laboratories. These factors can affect access to routine outpatient diagnostics, consultations, or the possibility of discharging ED children to “safety nets,” and therefore, they may have theoretically affected the pattern of ED access and consequently the prescription rates. To our knowledge, these non-clinical elements have never been widely including in previous studies. Interestingly, while we found no differences in the prescriptions for children evaluated during weekends, those assessed during night shifts had a lower odd of antibiotic prescription. Given the large samples, these data suggest that these factors should reasonably do not affect the prescription decisions, although a much smaller study from Malaysia assessing 500 children revealed a higher prescription rate during weekends [6].

Among factors not related to the patients’ conditions, the expertise of the treating physician could indeed affect the prescription likelihood. Interestingly, and contrary to common sense, physicians with a longer experience had a higher odds of prescribing antibiotics, including Am/Cl. This finding is interesting and may suggest that younger doctors may have more attention toward antimicrobial stewardship programs, whose importance has received more attention during recent years. For example, the WHO recently implemented the AWaRe (Access, Watch, and Reserve) classification to help allocate the proper choices of antibiotics, which may be more familiar to doctors closer to training periods [23, 24]. The fact that younger doctors traditionally make more night shifts may in part explain why children assessed during the night had a much lower probability of receiving antibiotics, confirmed in multivariate analyses.

Another important non-clinical factor is the contribution of temperature, and therefore seasons, on human health. There is increasing recognition that global climate change can directly affect health, and therefore, we included seasons of evaluation as a potential variable affecting probability of antibiotic prescription [25]. Overall, we found that children assessed in summer had lower rates of prescription (around 1 in 5) while those seen in winter the highest (around 1 in 3 children) (*p* 0.001), although in multivariate analyses, differences were statistically not significant. However, when the analyses were performed for age groups, those 2–5 years old had significantly higher probability to receive antibiotics in winter. Interestingly, this is the age group that is more affected by viral infections during school age. However, since climate change mainly refer to shifts of mean temperatures during seasons [25], a next aim for our research group is to address how specific conditions, and antibiotic prescriptions, are affected by significant changes in local temperatures from historic mean values, a parameter that we have not been able to address in current study.

The high rates of antibiotic prescriptions and the variability in practice [11] may reflect, somehow, fears of worsening conditions after discharging or missing bacterial infections [26], which may subsequently have repercussions on both the child, but on the discharging physician as well. However, there is growing evidence that for patients presenting with suspected bacterial infections, withholding antibiotic therapy may be acceptable in most cases unless septic shock or bacterial meningitis are suspected [27]. Even among children with suspected CAP, the outcomes are not statistically different between those who did and did not receive an antibiotic [8].

To further highlight the uncertainties behind the decision to prescribe antibiotics, a recent randomized clinical trial found the unexpected results that the use of rapid respiratory pathogen testing in the ED for children with influenza-like illness did not decrease antibiotic prescribing [28]. Although clear evidence of a viral infection should theoretically reinforce the decision to not prescribe antibiotics, in a cohort of children with respiratory symptoms with a high pre-test probability of having viral infections, previous studies found similar results on how these tests did not affect the prescription practices [29, 30]. In all these studies, authors concluded that antibiotic-prescribing decisions were likely made immediately following clinical evaluation, before results were available, as we also found in our cohort. These findings may reflect clinicians’ awareness about the complex relationship between bacterial and viral pathogens and their interplay in the definition of disease severity [31]. This novel concept probably better reflects the real-life scenarios that current biomarkers are not yet able to define, and available research has failed to address so far [19]. Finally, psychological factors may be an important, and more difficult to measure, element that can affect final decisions. For example, parental fears and pressures to receive antibiotics and their socioeconomic status may unconsciously impact physicians’ decisions [3]. However, to our knowledge, the psychological dynamics of this interplay has never been assessed through prospective studies.

Our study, being the largest one that included several variables in terms of demographic and clinical data, investigations, daytime variability, doctors’ experience, and decisions to perform diagnostics, provides strong confirmation of the complexity behind the decisions to prescribe or not antibiotics in the pediatric ED. Our data are in line with the recent PERFORM positions [19], strengthening the need for a new approach in fighting this problem. With the growing emergence of antibiotic resistance and its clear short- and long-term impact on global health, tackling antibiotic prescriptions is an urgent priority. However, focusing on a single aspect of the problem, for example on biomarkers, will not provide benefits, while it is becoming more evident that more comprehensive strategies are needed. Pro-active patients/parents’ involvement, proper antimicrobial stewardship programs focused on the specificity of ED settings, new biomarkers, and wider availability of rapid microbiological diagnostic tests could favorably influence the clinical practice. However, also an improved definition of confirmed/probable viral/bacterial infections, a deeper focus on factors associated with clinical presentations, and physicians’ feelings are each necessary factors to be incorporated in real-world settings and research projects.

Our study has some limitations to address. The retrospective nature of the study is an intrinsic limitation, although our electronic system allowed us to include and analyze a large number of detailed variables. Second, in our institution, the use of procalcitonin has been introduced late in 2020, and its use is not yet considered routine in our internal guidelines. Third, we have not been able to collect data on the length of antibiotic therapies prescribed. Finally, we did not include the immunizations performed by the patient at the moment of admission. Theoretically, the knowledge of immunization status may lower the risk of serious infection on discharge and influence the antibiotic prescription. However, since the overall coverage in Italian children is above 90% in our region for most immunizations [32], this data is not expected to have had a major impact on prescriptions. Last, we do not have data about microbiological data. However, since we included only children discharged from the ED, in our setting, it is uncommon to perform microbiological studies in children that are planned to be discharged directly from the ED without being admitted.

In conclusions, our analysis confirmed that the children discharged from the pediatric ED receive a high rate of antibiotic prescriptions, most of the time with Am/Cl. The inclusion of several non-clinical variables allowed us to explore the complexity of the reasons behind the pediatricians’ decision of prescribing antibiotics, highlighting the importance of developing a new and comprehensive approach to improve antibiotic use in the ED. This approach should include the simultaneous participation of all actors involved in this process: doctors, parents, children, antimicrobial stewardship programs, diagnostics, and a new categorization of viral and bacterial infections.

## Supplementary Information

Below is the link to the electronic supplementary material.

Supplementary file1 (DOCX 1014 KB)

## Data Availability

Available upon request to the corresponding author.

## Abbreviations

Am/Cl: Amoxicilline/clavulanate
CXR: Chest X-ray
