# Supplementary material for: Determinants of antibiotic prescriptions in a large cohort of children discharged from a pediatric emergency department
Source: Eur J Pediatr. 2022 Feb 4;181(5):2017–30. doi: 10.1007/s00431-022-04386-y (PMC8813572; doi:10.1007/s00431-022-04386-y)
Supplement: Supplementary file 1 — Supplementary file1 (DOCX 1014 KB) [file 431_2022_4386_MOESM1_ESM.docx]

**Supplementary material**

| **Variable** | **Age group < 1 year Odds Ratio**  **(95% Confidence Interval)** | **Age group 2 - 5 years Odds Ratio**  **(95% Confidence Interval)** | **Age group 6 - 10 years Odds Ratio**  **(95% Confidence Interval)** | **Age group 11 - 18 years Odds Ratio**  **(95% Confidence Interval)** |
| --- | --- | --- | --- | --- |
| *Ed Presentation* |  |  |  |  |
| Season of ED access |  |  |  |  |
| - Summer | Reference | Reference | Reference | Reference |
| - Fall | 1.13 (0.94 – 1.37) | 1.07 (0.97 – 1.18) | **0.79 (0.67 – 0.93)*** | **0.71 (0.58 – 0.88)*** |
| - Winter | 1.13 (0.95 – 1.35) | **1.23 (1.12 – 1.34)**** | **0.78 (0.67 – 0.91)**** | 0.88 (0.73 – 1.05) |
| - Spring | 1.18 (0.98 – 1.42) | **1.10 (1.00 – 1.21)*** | **0.83 (0.72 – 0.97)*** | **0.73 (0.60 – 0.89)*** |
| Access on Weekend | 0.91 (0.81 – 1.02) | **0.94 (0.88 – 0.99)*** | 1.02 (0.92 – 1.13) | 1.14 (0.98 – 1.32) |
| Access on nightshift | **0.87 (0.78 – 0.97)*** | **0.82 (0.77 – 0.87)**** | **0.85 (0.77 – 0.95)*** | **0.82 (0.72 – 0.95)*** |
| Pediatrician expertise >3 years | **0.76 (0.63 – 0.91)*** | **0.86 (0.78 – 0.96)**** | 0.98 (0.83 – 1.17) | **4.37 (3.58 – 5.33)**** |
| *Reported Symptoms* |  |  |  |  |
| Cough | 0.92 (0.81 – 1.03) | 0.94 (0.88 – 1.01) | **0.85 (0.74 – 0.97)*** | **0.85 (0.74 – 0.97)*** |
| Vomit | 0.95 (0.82 – 1.11) | **0.83 (0.76 – 0.89)**** | **0.81 (0.70 – 0.93)*** | **0.81 (0.70 – 0.93)*** |
| Dyspnea | **0.52 (0.41 – 0.66)**** | **0.65 (0.55 – 0.77)**** | **0.47 (0.32 – 0.68)**** | **0.47 (0.32 – 0.68)**** |
| Diarrhea | 1.06 (0.88 – 1.27) | 1.01 (0.90 – 1.12) | 0.91 (0.71 – 1.16) | 0.91 (0.71 – 1.16) |
| Fever peak > 40°C | **1.80 (1.12 – 2.01)*** | **1.23 (1.06 – 1.43)*** | 1.32 (0.96 – 1.83) | 1.32 (0.96 – 1.83) |
| Myalgia/Arthralgia | 1.00 (0.99 – 1.01) | 1.06 (0.48 – 2.35) | 0.61 (0.27 – 1.38) | 0.61 (0.27 – 1.38) |
| *Clinical Evaluation* |  |  |  |  |
| Fever > 37.5°C on admission | **1.93 (1.65 – 2.27)**** | **1.23 (1.06 – 1.43)*** | **1.35 (1.20 – 1.52)**** | **1.20 (1.03 – 1.41)*** |
| Crepitation on auscultation | **1.95 (1.75 – 2.17)**** | **2.03 (1.75 – 2.36)**** | **1.87 (1.41 – 2.48)**** | **1.85 (1.13 – 3.04)*** |
| Poor/mediocre general condition | **1.84 (1.44 – 2.35)**** | **1.59 (1.22 – 2.07)**** | 1.58 (0.92 – 2.70) | 1.59 (0.84 – 3.01) |
| Peripheral cyanosis | 0.74 (0.42 – 1.32) | 0.86 (0.61 – 1.21) | 0.68 (0.36 – 1.28) | 0.74 (0.35 – 1.54) |
| *Laboratory and radiology* |  |  |  |  |
| PCR Categoric   - Not requested - < 50 mg/dl - ≥ 50 mg/dl | Reference 1.38 (0.68 – 2.78) 2.39 (0.91 – 6.25) | Reference **1.51 (1.01 – 2.26)*** **3.75 (2.28 – 6.17)**** | Reference 1.39 (0.74 – 2.59) **4.06 (1.88 – 8.75)**** | Reference 1.59 (0.94 – 2.68) **3.12 (1.64 – 5.94)**** |
| WBC Categoric   - Not requested - < 10k cell/mm3 - ≥ 10k cell/mm3 | Reference 0.99 (0.46 – 2.10) 1.47 (0.70 – 3.11) | Reference 0.78 (0.51 – 1.21) 1.13 (0.74 – 1.73) | Reference 0.69 (0.37 – 1.31) 1.04 (0.54 – 2.01) | Reference 0.57 (0.40 – 1.17) 1.24 (0.72 – 2.14) |
| Urine analysis   - Not requested - Negative - Positive | Reference **2.19 (1.36 – 3.51)**** 1.07 (0.48 – 2.35) | Reference 1.15 (0.72 – 1.85) 1.61 (0.82 – 3.13) | Reference 0.97 (0.38 – 2.48) 2.30 (0.83 – 6.39) | Reference 0.34 (0.09 – 1.19) 1.61 (0.82 – 3.16) |
| Chest X Ray   - Not Requested - Negative - Positive | Reference **4.31 (3.20 – 5.81)** 16.84 (7.88 – 36.00)**** | Reference **1.67 (1.42 – 1.97)** 3.41 (2.53 – 4.59)**** | Reference **1.57 (1.17 – 2.11)** 3.33 (2.14 – 5.18)**** | Reference 0.91 (0.66 – 1.27) **1.83 (1.04 – 3.28)*** |
| *Clinical history* |  |  |  |  |
| Inherited disease | 1.05 (0.96 – 1.28) | 1.33 (0.88 – 2.00) | 1.57 (0.79 – 3.10) | 1.07 (0.57 – 2.01) |
| History of cardiac disease | 0.97 (0.44 – 1.15) | **5.82 (1.03 – 33.00)*** | 0.85 (0.12 – 5.79) | 2.23 (0.51 – 9.70) |
| *Diagnostic Group* |  |  |  |  |
| Upper airway infections | **3.99 (3.46 – 4.59)**** | **4.08 (3.78 – 4.39)**** | **4.99 (4.38 – 5.70)**** | **5.95 (4.93 – 7.19)**** |
| Lower airway infections | **2.31 (1.87 – 2.86)**** | **6.67 (5.87 – 7.58)**** | **10.05 (8.01 – 12.61)**** | **11.20 (8.02 – 15.63)**** |
| Abdominal | **0.16 (0.06 – 0.27)**** | **0.13 (0.09 – 0.18)**** | **0.16 (0.11 – 0.25)**** | **0.25 (0.16 – 0.41)**** |
| Urinary tract infections | **12.26 (8.84 – 17.01)**** | **11.55 (9.19 – 14.52)**** | **14.64 (10.76 – 19.92)**** | **9.83 (6.97 – 13.87)**** |
| Cutaneous | **2.27 (1.53 – 3.38)**** | **4.87 (4.14 – 5.75)**** | **7.57 (5.89 – 9.71)**** | **7.83 (5.68 – 10.79)**** |
| Other specified | 0.78 (0.24 – 2.55) | **1.31 (1.02 – 1.68)*** | **1.74 (1.22 – 2.48)*** | 1.21 (0.77 – 1.89) |
| Fever (unspecified) | Reference | Reference | Reference | Reference |

** p value <0.01; ** p value <0.001*

**Supplementary Table 1.** Multivariate logistic regression analysis of factors associated with an antibiotic prescription at Emergency Department discharge. The logistic models are carried out separately for each age group. Constant is included in all models. The analysis was carried out only including significant factors at univariate analysis.

| **Variable** | **Other  Antibiotic  (n 11221)** | **Amoxicillin  (n 1946)** | **Univariate p  value** | **Odds Ratio  for  Prescription** | **Multivariate p value** |
| --- | --- | --- | --- | --- | --- |
| Sex (male) | 6237 (55.6) | 1090 (56.0) | 0.725 |  |  |
| Age group |  |  |  |  | **<0.001** |
| - < 1 year | 1304 (11.6) | 498 (25.6) |  | Reference |  |
| - 2-5 years | 6174 (55.0) | 1167 (60.0) | **<0.001** | 0.46 [0.40 – 0.53] | **<0.001** |
| - 6-10 years | 2440 (21.7) | 232 (11.9) |  | 0.25 [0.21 – 0.30] | **<0.001** |
| - 11-18 years | 1303 (11.6) | 49 (2.5) |  | 0.10 [0.08 – 0.14] | **<0.001** |
| *Ed Presentation* |  |  |  |  |  |
| Triage Code   - Emergency - Urgent - Non Urgent - Ambulatory | 10 (0.1)  673 (6.1)  10330 (93.4)  47 (0.4) | 2 (0.1)  80 (4.3)  1786 (95.4)  5 (0.3) | **0.013** | Reference 0.21 [0.42 – 1.04]  0.24 [0.49 – 1.17] 0.17 [0.27 – 1.09] | 0.196  0.056 0.078 0.062 |
| Access on Weekend | 4259 (38.0) | 812 (41.7) | **0.002** | 1.11 [0.99 – 1.23] | 0.055 |
| Season of ED access |  |  |  |  |  |
| - Summer | 1873 (16.7) | 297 (15.3) |  |  |  |
| - Fall | 2639 (23.5) | 458 (23.5) | 0.453 |  |  |
| - Winter | 3947 (35.2) | 703 (36.1) |  |  |  |
| - Spring | 2762 (24.6) | 488 (25.1) |  |  |  |
| Access on nightshift | 4956 (44.2) | 915 (47.0) | **0.019** | 1.07 [0.96 – 1.18] | 0.226 |
| Access by EMS | 283 (2.5) | 42 (2.2) | 0.340 | 1.41 [1.09 – 1.80] | **0.008** |
| Pediatrician expertise >3 years | 10413 (92.8) | 1556 (80.0) | **<0.001** | 0.27 [0.23 – 0.31] | **<0.001** |
| *Reported Symptoms* |  |  |  |  |  |
| Cough | 3794 (33.8) | 780 (40.1) | **<0.001** | 1.34 [1.20 – 1.50] | **<0.001** |
| Vomit | 1538 (13.7) | 321 (16.5) | **0.001** | 1.11 [0.96 – 1.29] | 0.140 |
| Dyspnea | 412 (3.7) | 44 (2.3) | **0.002** | 0.69 [0.49 – 0.97] | **0.034** |
| Diarrhea | 797 (7.1) | 200 (10.3) | **<0.001** | 1.08 [0.89 – 1.29] | 0.433 |
| Fever peak > 40°C | 424 (3.8) | 83 (4.3) | 0.303 |  |  |
| Myalgia/Arthralgia | 20 (0.2) | 2 (0.1) | 0.762 |  |  |
| Palpitations | 25 (0.2) | 2 (0.1) | 0.416 |  |  |
| Headache | 321 (2.9) | 30 (1.5) | **0.001** | 0.90 [0.61 – 1.34] | 0.618 |
| *Clinical Evaluation* |  |  |  |  |  |
| Fever > 37.5°C on admission | 2287 (20.4) | 275 (14.1) | **<0.001** | 1.16 [1.01 – 1.36] | **0.042** |
| Crepitation on auscultation | 936 (8.3) | 1.6 (5.4) | **<0.001** | 0.92 [0.73 – 1.17] | 0.520 |
| Poor or mediocre general condition | 186 (1.7) | 27 (1.4) | 0.383 |  |  |
| Peripheral cyanosis | 88 (0.8) | 8 (0.4) | 0.074 |  |  |
| *Laboratory and radiology* |  |  |  |  |  |
| PCR Categoric   - Not requested - < 50 mg/dl - ≥ 50 mg/dl | 10422 (92.9) 574 (5.1) 225 (2.0) | 1861 (95.6) 63 (3.2) 22 (1.1) | **<0.001** | Reference 0.81 [0.42 – 1.58] 0.73 [0.34 – 1.61] | 0.542 0.443 |
| WBC Categoric   - Not requested - < 10k cell/mm3 - ≥ 10k cell/mm3 | 10435 (93.0)  319 (2.8)  467 (4.2) | 1862 (95.7)  35 (1.8)  49 (2.5) | **<0.001** | Reference 1.01 [0.49 – 2.05] 0.93 [0.47 – 1.85] | 0.975 0.842 |
| Urine analysis   - Not requested - Negative - Positive | 11060 (98.6)  63 (0.6)  98 (0.9) | 1937 (99.5)  9 (0.5)  0 | **<0.001** | Reference 0.79 [0.38 – 1.62] / | 0.521 0.996 |
| Chest X Ray   - Not Requested - Negative - Positive | 10117 (90.2)  629 (5.6)  475 (4.2) | 1864 (95.8)  64 (3.3)  18 (0.9) | **<0.001** | Reference  0.69 [0.52 – 0.93]  0.35 [0.21 – 0.58] | **0.014**  **<0.001** |
| *Clinical history* |  |  |  |  |  |
| Inherited disease | 91 (0.8) | 9 (0.5) | 0.102 |  |  |
| History of cardiac disease | 14 (0.1) | 0 | 0.247 |  |  |
| Prematurity | 69 (0.6) | 11 (0.6) | 0.795 |  |  |
| History of asthma | 347 (3.1) | 41 (2.1) | **0.018** | 0.79 [0.64 – 0.98] | **0.039** |
| Intellectual disability | 11 (0.1) | 1 (0.1) | 1.000 |  |  |
| History of epilepsy | 48 (0.4) | 6 (0.3) | 0.447 |  |  |
| *Diagnostic Group* |  |  |  |  |  |
| Upper airway infections | 6125 (54.6) | 1380 (70.9) | **<0.001** | 1.61 [1.38 – 1.88] | **<0.001** |
| Lower airway infections | 2026 (18.1) | 193 (9.9) |  | 0.82 [0.64 – 1.03] | 0.090 |
| Abdominal | 70 (0.6) | 12 (0.6) |  | 1.77 [0.90 – 3.45] | 0.097 |
| Urinary tract infections | 593 (5.3) | 24 (1.2) |  | 0.36 [0.23 – 0.57] | **<0.001** |
| Cutaneous | 518 (4.6) | 65 (3.3) |  | 1.14 [0.83 – 1.55] | 0.414 |
| Other specified | 142 (1.3) | 14 (0.7) |  | 1.07 [0.60 – 1.92] | 0.802 |
| Fever (unspecified) | 1747 (15.6) | 258 (13.3) |  | Reference |  |

**Supplementary Table 2** – Factors associate to the choice of Amoxicillin prescription at discharge. Univariate and Multivariate analysis were reported considering only the 13.167 patients discharged with an antibiotic prescription.

**Supplementary figure S1**


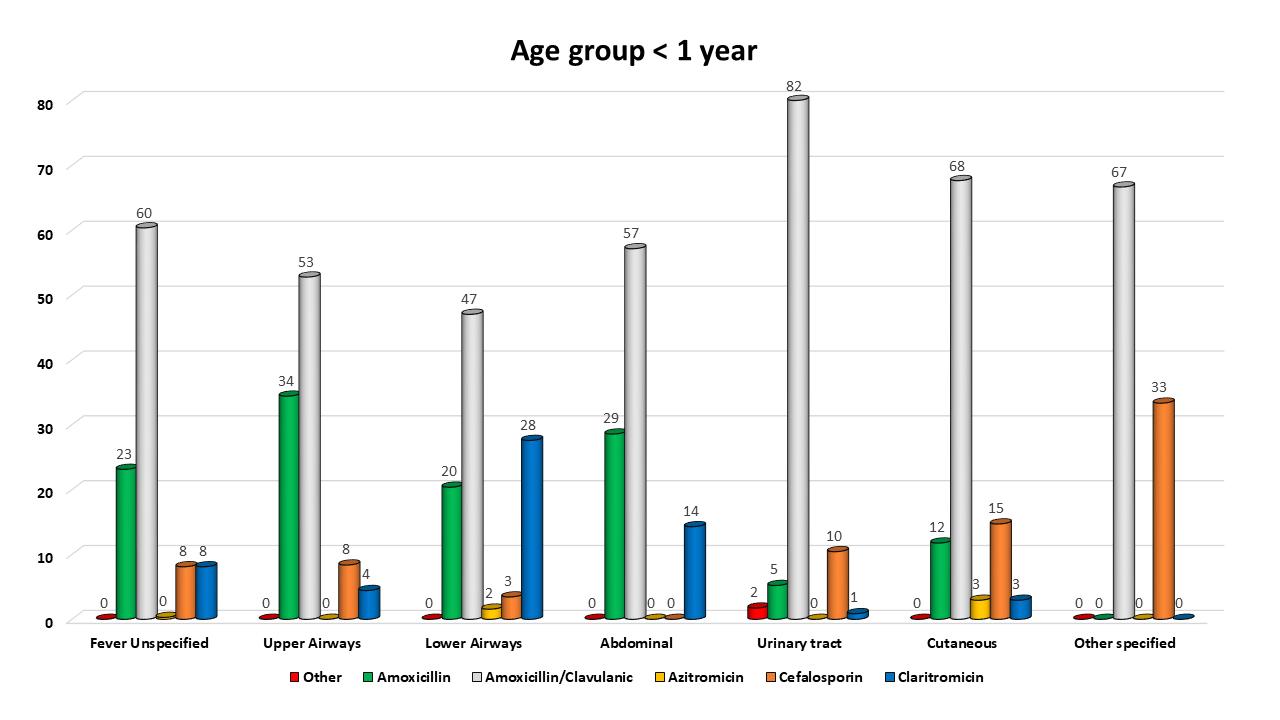


**Supplementary figure S2**

**
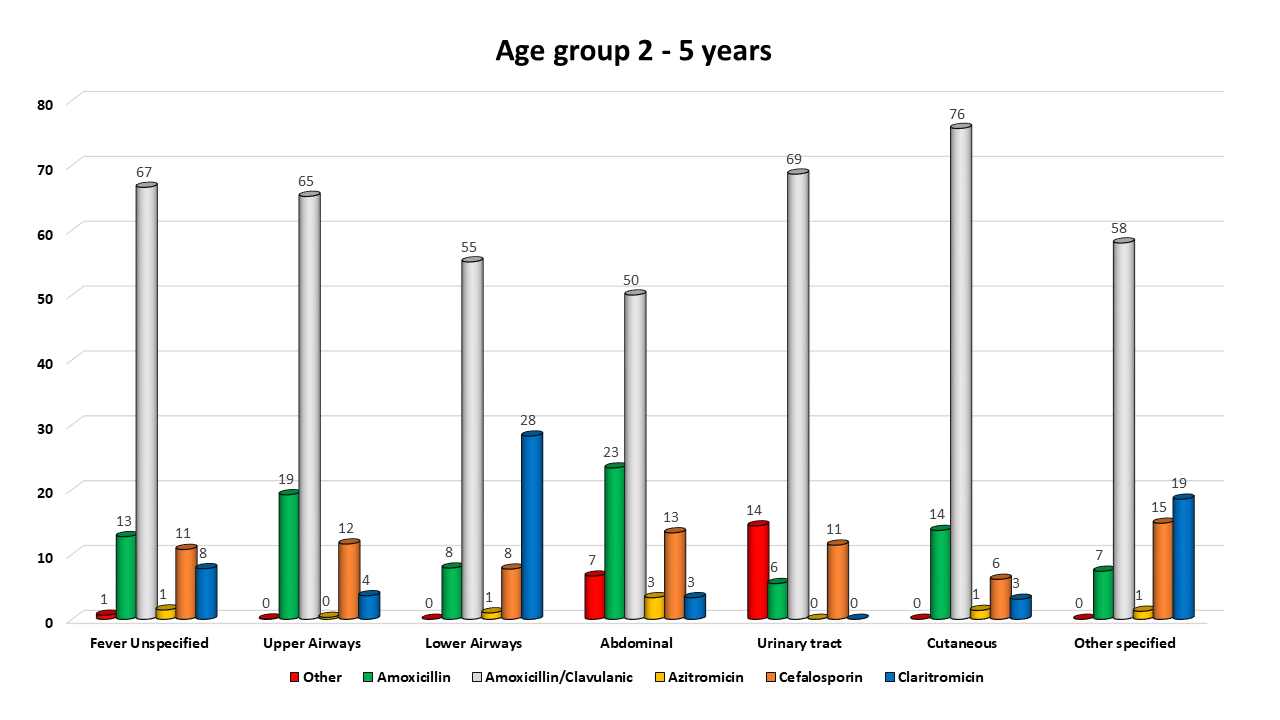
**

**Supplementary figure S3**

**
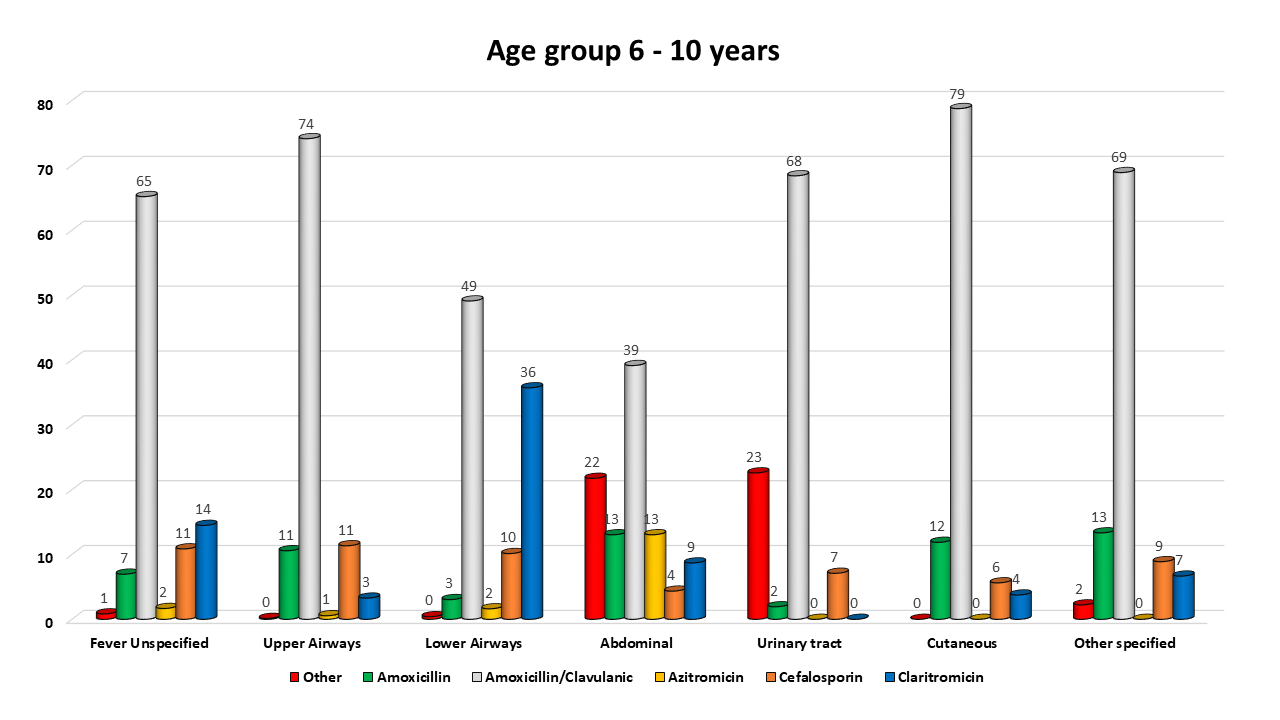
**

**Supplementary figure S4**

**
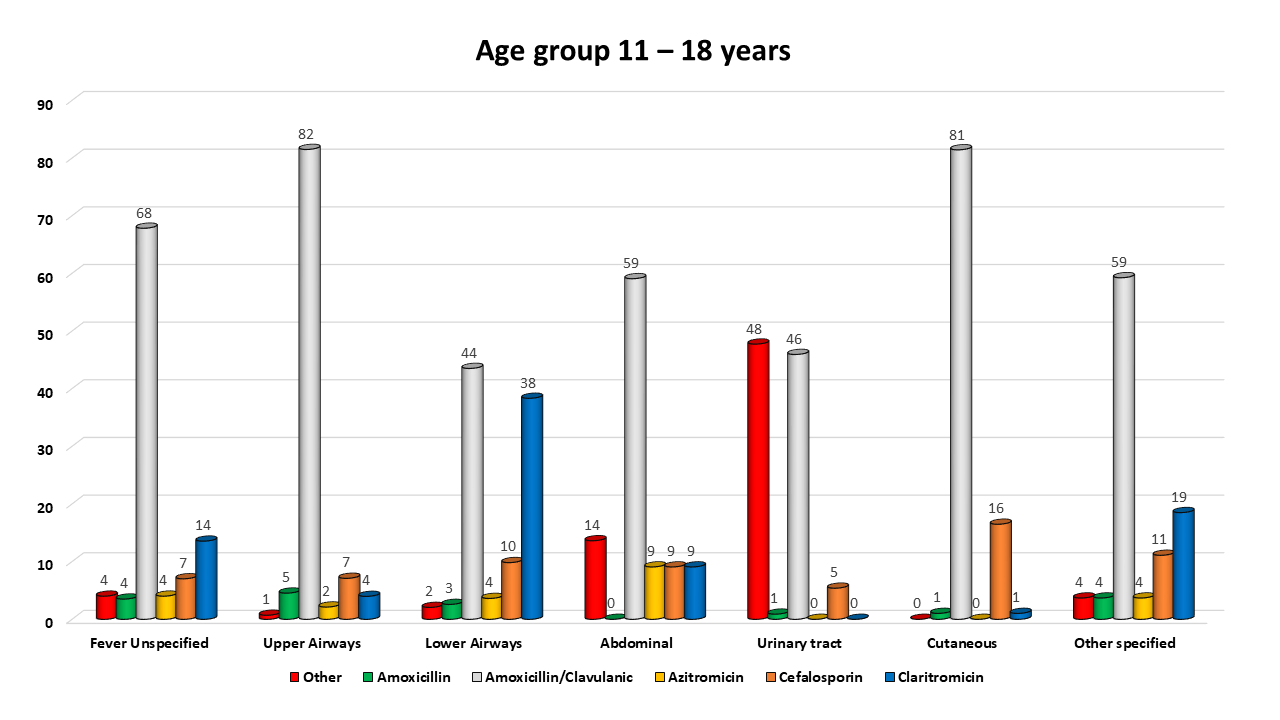
**
